# Dosimetric comparison of distal esophageal carcinoma plans for patients treated with small‐spot intensity‐modulated proton versus volumetric‐modulated arc therapies

**DOI:** 10.1002/acm2.12623

**Published:** 2019-05-21

**Authors:** Chenbin Liu, Ronik S. Bhangoo, Terence T. Sio, Nathan Y. Yu, Jie Shan, Jennifer S. Chiang, Julia X. Ding, William G. Rule, Shawn Korte, Pedro Lara, Xiaoning Ding, Martin Bues, Yanle Hu, Todd DeWees, Jonathan B. Ashman, Wei Liu

**Affiliations:** ^1^ Department of Radiation Oncology Mayo Clinic Phoenix AZ 85054 USA; ^2^ Division of Biostatistics Mayo Clinic Phoenix AZ 85054 USA

**Keywords:** distal esophageal, intensity‐modulated proton therapy, interplay effects, small spot size, volumetric‐modulated arc therapy

## Abstract

**Background:**

Esophageal carcinoma is the eighth most common cancer in the world. Volumetric‐modulated arc therapy (VMAT) is widely used to treat distal esophageal carcinoma due to high conformality to the target and good sparing of organs at risk (OAR). It is not clear if small‐spot intensity‐modulated proton therapy (IMPT) demonstrates a dosimetric advantage over VMAT. In this study, we compared dosimetric performance of VMAT and small‐spot IMPT for distal esophageal carcinoma in terms of plan quality, plan robustness, and interplay effects.

**Methods:**

35 distal esophageal carcinoma patients were retrospectively reviewed; 19 patients received small‐spot IMPT and the remaining 16 of them received VMAT. Both plans were generated by delivering prescription doses to clinical target volumes (CTVs) on phase‐averaged 4D‐CT's. The dose‐volume‐histogram (DVH) band method was used to quantify plan robustness. Software was developed to evaluate interplay effects with randomized starting phases for each field per fraction. DVH indices were compared using Wilcoxon rank‐sum test. For fair comparison, all the treatment plans were normalized to have the same CTV_high_ D_95%_ in the nominal scenario relative to the prescription dose.

**Results:**

In the nominal scenario, small‐spot IMPT delivered statistically significantly lower liver D_mean_ and V_30Gy[RBE]_, lung D_mean_, heart D_mean_ compared with VMAT. CTV_high_ dose homogeneity and protection of other OARs were comparable between the two treatments. In terms of plan robustness, the IMPT and VMAT plans were comparable for kidney V_18Gy[RBE]_, liver V_30Gy[RBE]_, stomach V_45Gy[RBE]_, lung D_mean_, V_5Gy[RBE]_, and V_20Gy[RBE]_, cord D_max_ and $D_{0.03cm^{3}}$, liver D_mean_, heart V_20Gy[RBE]_, and V_30Gy[RBE]_, but IMPT was significantly worse for CTV_high_ D_95%_, $D_{2cm^{3}}$, and D_5%_‐D_95%_, CTV_low_ D_95%_, heart D_mean_, and V_40Gy[RBE]_, requiring careful and experienced adjustments during the planning process and robustness considerations. The small‐spot IMPT plans still met the standard clinical requirements after interplay effects were considered.

**Conclusions:**

Small‐spot IMPT decreases doses to heart, liver, and total lung compared to VMAT as well as achieves clinically acceptable plan robustness. Our study supports the use of small‐spot IMPT for the treatment of distal esophageal carcinoma.

## 1 INTRODUCTION

Esophageal carcinoma is the eighth most common cancer and the sixth most common cause of cancer deaths worldwide. There are an estimated 17 290 new cases and 15 850 deaths annually in America, and distal esophageal cancer cases are increasing rapidly in developed countries.^1^, ^2^ In recent years, trimodality therapy (neoadjuvant chemoradiation followed by esophagectomy) has improved clinical outcomes in patients with locally advanced esophageal cancers compared to surgery alone.^3^, ^4^ Concurrent chemotherapy, usually with weekly carboplatin and paclitaxel, combined with radiation doses of 41.4–50.4 Gy are considered standard treatments in the modern era.^5^ The long‐term results of Radiation Therapy Oncology Group (RTOG) clinical trial 85‐01 confirmed that chemoradiation increased overall survival for patients with esophageal carcinoma compared with radiotherapy (RT) alone.^5^ Due to proximity to surrounding organs at risk (OAR) such as heart, spinal cord, lungs, kidney, liver, and the remaining stomach, the RT planning for distal esophagus carcinoma poses special challenges.^6^, ^7^ Sufficient radiation doses must be applied to the tumor and lymph node areas, while protecting nearby critical normal structures.

Volumetric‐modulated arc therapy (VMAT) is an advanced form of intensity‐modulated radiation therapy (IMRT) that can deliver a highly conformal dose distribution using single or multiple arcs.^8^ Compared with static‐field IMRT, VMAT achieves similar OAR sparing and planning target volume (PTV) coverage with significantly shorter treatment time.^9^, ^10^ Proton beam therapy delivers highly conformal target coverage, while sparing adjacent OARs due to its unique Bragg peak dose deposition characteristics. Proton beam therapy^11^ has several forms of delivery including passive scattering (PSPT), uniform scanning, and intensity‐modulated proton therapy (IMPT). Unlike PSPT, IMPT uses magnetic steering of a narrow proton beam, termed a *beamlet*, to deliver a modulated dose to a spot of a specified size, which offers improved high‐dose conformality compared with PSPT and better OAR sparing in the mid‐ to low‐dose range compared to IMRT.^12^, ^13^ Therefore, it is hypothesized that IMPT can improve the therapeutic ratio which will result in fewer adverse effects, while achieving the same tumor control as IMRT or better.^14^

However, IMPT is highly sensitive to setup and range uncertainties, as well as vulnerable to respiratory motion present in the distal esophageal regions.^15^, ^16^ The uncertainties originate from daily patient alignment, conversion of Hounsfield units to stopping power, artifacts in computed tomography, and anatomical changes in patients etc.^17^ The interaction between dynamic beamlet delivery and respiratory motion, also called interplay effect, may degrade the quality of planned dose distributions,^18^, ^19^, ^20^, ^21^, ^22^, ^23^, ^24^, ^25^, ^26^, ^27^, ^28^, ^29^, ^30^ compromising the safety and efficacy of the proposed treatment. In addition, large spot sizes, common in older IMPT machines, tend to lead to larger penumbras, which results in undesired dose to adjacent OARs. The majority of new proton facilities offer smaller spot sizes, which can produce smaller penumbras and better OAR sparing. However, smaller spot‐size plans can exacerbate the negative consequences of patient setup uncertainty and make interplay effects more prominent.^25^, ^31^ As a result, we first need to quantify and then mitigate the impact of uncertainties and interplay effects for small‐spot IMPT for the treatment of distal esophageal cancers.

Previous studies have focused on the comparison of plan quality alone among plans generated using either PSPT, large‐spot IMPT (spot size σ as large as 6–15 mm depending on proton energies), and/or IMRT^32^, ^33^, ^34^, ^35^, ^36^, ^37^ with no mention of plan uncertainties or motion effect analyses. Recently, Shiraishi et al. reported that in a large cohort of esophageal cancer patients, PSPT and large‐spot IMPT significantly reduced radiation exposures to the whole heart and cardiac substructures compared with IMRT.^32^ Welsh et al. found that large‐spot IMPT for distal esophageal carcinoma also lowered the doses to bilateral lungs and liver compared to IMRT.^37^ More recently, a large, retrospective multi‐institutional study also demonstrated that proton beam therapy appeared to be more clinically advantageous compared with 3‐dimensionl conformal RT and IMRT in lowering the incidence of pulmonary and cardiac complications as well as the mean length of in‐hospital stay.^38^

To the best of our knowledge, no dosimetric study has been reported comparing plan quality and plan robustness for small‐spot IMPT and VMAT in the treatment of distal esophageal cancer. The aim of this study is to evaluate the feasibility of small spot IMPT for such treatments. We compared plan quality and robustness for VMAT and small‐spot IMPT. The interplay effects of small‐spot IMPT were also quantified.

## 2 MATERIALS AND METHODS

### 2.1 Patient selection

Nineteen consecutive patients with distal esophageal carcinoma treated with IMPT and 16 patients treated with VMAT between May 2014 and September 2017 at our institution were retrospectively reviewed. Table 1 shows the patient characteristics for IMPT and VMAT treatment groups. The patients included in this study were not randomly selected, but were carefully selected by an experienced physicist from the existing database to ensure that the patients from the two treatment groups did not show significant differences in age, gender, body mass, tumor volumes, motion amplitude, or prescription dose (Table 1); the cases were consecutively considered based on radiotherapy treatment start date. Patients were excluded from this study if they were less than 18 yr old and/or not treated with curative intent. All treatment plans included in this work were approved and delivered clinically. Patients were staged using PET/CTs. All patients had an Eastern Cooperative Oncology Group performance status of 2 or better. No patients in this study had implanted cardiac devices.

**Table 1:** Patient characteristics between intensity‐modulated proton therapy (IMPT) and volumetric‐modulated arc therapy (VMAT) treatment groups.

	IMPT	VMAT	*P*‐value
Patient number	19	16	
Age at treatment (yr)			0.79
Median (range)	71 (54–84)	71 (55–83)	
Gender			0.42*
Male, No. (%)	14 (73.7)	14 (87.5)	
Body mass (kg)			0.55
Median (range)	83.2 (53.8–123.0)	82.5 (69.9–111.3)	
CTV_high_ volume (cm^3^)			0.40
Median (range)	205.0 (117.6–515.6)	219.0 (102.5–531.4)	
CTV_low_ volume (cm^3^)			0.98
Median (range)	440.00 (151.8–1144.7)	447.10 (140.9–773.8)	
Motion amplitude (mm)			0.91
Median (range)	8 (4–13)	6.2 (6–13.7)	

CTV, clinical target volume.

* *P*‐value from Fisher's exact test. Others are *P*‐values from Wilcoxon rank‐sum test.

### 2.2 Patient simulation and contouring

For both IMPT and VMAT treatments, the processes for patient simulation and contouring were similar. Four‐dimensional computed tomography (4D‐CT) was used to simulate all patients in the supine position. At our clinic, we defined the respiratory motion amplitude by the largest displacement of tumor geometric center in the anterior–posterior (A‐P), superior–inferior (S‐I), and right–left (R‐L) directions in all 4D breathing‐phase CTs. Most commonly, the S‐I direction demonstrated the largest motion amplitude. Immobilization devices including wing board (CIVCO, Kalona, IA) and vacuum bag (Elekta, Atlanta, GA) were used for all patients during 4D CT simulation and daily treatments.

The commercial treatment planning system (Eclipse^TM^, version 13, Varian medical system, Palo Alto, CA) was used to generate treatment plans based on the simulation 4D CTs, which were used to localize the targets and OARs. Heart, cord, liver, stomach, bowel, and kidney and targets were contoured on the 4D‐averaged CT. The same clinical target volumes (CTV_high_ and CTV_low_) were used for IMPT and VMAT treatment planning. The prescription doses for CTV_high_ and CTV_low_ were 50.4 and 45.0 Gy[RBE] or 50.0 and 45.0 Gy[RBE] with simultaneously integrated boost, respectively; 41.4 Gy[RBE] was also allowed for CTV_low_ or if the patient was treated preoperatively. CTV_low_ and CTV_high_ were generated as follows: first, we identified the appropriate gross target volume (GTV) on the average CT or one of the respiratory phase CT scans, and the co‐registered PET and CT scan. Then, a 3–4 cm expansion was added along the mucosal surface longitudinally, in addition to a 1–1.2 cm radial expansion for the CTVs which were anatomically constrained. The lower CTV volume typically included a small expansion of elective nodal volumes in the para‐esophageal region. The treating radiation oncologist also adjusted the expansion of margins based on the pathology and location of the tumor; the potential microscopic tumor extent and anatomic boundaries of heart, lungs, liver, kidneys, and bowel were also taken into consideration in the final target delineation.

### 2.3 Treatment planning

In VMAT treatment planning, all treatment plans were generated on the averaged CT. We used planning target volumes (PTV), formed by a 5‐mm uniform expansion of CTVs, for plan optimization and evaluation in VMAT. Most commonly, 2 to 3 arcs were used. Photon optimizer (PO) model in the Eclipse^TM^ TPS was used for VMAT optimization, and analytical anisotropic algorithm (AAA) model was used for dose calculation. The dose calculation grid size was 3 mm. The dosimetrists created treatment plans, which satisfied institutional dose constraints (Table 2) for OAR sparing. For target coverage, V_100%_ of PTV_high_ was at least 95% and $D_{0.03cm^{3}}$ of PTV_high_ was no more than 110% of prescription dose.

**Table 2:** Dose volume constraints for organs‐at‐risk for esophageal carcinoma treatments.

Structure	Dose limits (Gy[RBE])
Liver	D_mean_ < 25 Gy[RBE]; V_30Gy[RBE]_ < 60%
Total lung	V_5Gy[RBE]_ < 60%; V_20Gy[RBE]_ < 15%; D_mean_ < 15 Gy[RBE]
Spinal cord	D_max_ < 45 Gy[RBE]; V_45Gy[RBE]_ < 0.1%
Heart	V_25Gy[RBE]_ < 50%; V_40Gy[RBE]_ < 30%; D_mean_ < 26 Gy[RBE]
Left/right kidney	V_18Gy[RBE]_ < 10%; D_mean_ < 18 Gy[RBE]

In IMPT treatment planning, all treatment plans were also generated on the averaged CT. An optimization target volume (OTV) was formed by uniform expansion of the CTV by 5 mm to help generate a robust plan. Proton spots were placed strategically outside of the OTV as well to ensure homogenous dose distributions in the OTV. Usually, 2 to 3 proton beams were used. Single field optimization (SFO) was always the first option, however, multiple‐field optimization (MFO) was used if the plan generated by SFO did not meet the clinical requirements. For optimization, the pencil beam convolution supposition (PCS) model was used for SFO whereas the nonlinear uniform proton optimizer (NUPO) robust optimization was used for MFO in the Eclipse^TM^ TPS to generate a robust MFO plan.^39^, ^40^, ^41^, ^42^, ^43^ PCS model was used for the final dose calculation and beam line modifiers for all IMPT plans. The dose calculation grid size was 2.5 mm. Dosimetrists and medical physicists chose beam angles to minimize the impact of motion and spare normal tissues. Density override (HU = 50) was used for the CTV in IMPT treatment planning to help generate plans robust to respiratory motion.^30^, ^44^, ^45^ After the plan was generated on the averaged CT, the dosimetrists created two verification dose distributions without the density override by recalculating the dose distributions on the maximum exhale and maximum inhale phases of the 4D CT. This allowed evaluation of the impact of respiratory motion. The original plan would be adjusted until the dose distributions calculated on the averaged CT, maximum exhale, and maximum inhale phases without density override all met required dose volume constraints (Table 2), plan robustness quantification thresholds, and the prescription criteria (see Robustness Quantification subsection). The dosimetrists were allowed to override the beam path around the diaphragm region to compensate for the change of water equivalent thickness due to respiratory motion. By adjusting the size of override region and its density values, the verification plans allowed for improvement while the original plan still met the clinical criterion.

The treating radiation oncologist approved the final treatment plan after careful evaluation of plan quality, plan robustness, and interplay effects. Plan robustness and interplay effect were only evaluated for IMPT planning (See details in Plan Quality Evaluation, Robustness Quantification, and Interplay Effects Evaluation subsections below). In addition, a second independent dose calculation was performed using an in‐house developed Monte Carlo software.^46^ We carefully commissioned our TPS by fudging the proton optics parameters,^47^ so the dose distributions calculated by our TPS matched well with those generated by the Monte Carlo simulations.^47^, ^48^ Based on 3 yr clinical experience at our institution, the deviation of CTV D_95%_ and D_mean_ between Monte Carlo simulation and TPS computation has been less than 3% and 2% for most of patients respectively.

### 2.4 Treatment delivery

The Clinac machines (Varian medical system, Palo Alto, CA) were used to deliver the VMAT plans. The related parameters including field information, energy, and estimated delivery duration are shown in Table S1. Typically, daily on‐board imaging or cone‐beam CT was used as the image‐guided RT methods.

For IMPT, the Hitachi ProBeat‐V spot‐scanning proton beam machines (Hitachi, Tokyo, Japan) were used. The active scanning proton beam machine was commissioned to have an energy‐dependent spot size (σ) of 2 to 6 mm, with a fixed spot spacing of 5 mm. Our proton beam scanning machine had discrete proton energies ranging from 71.3 to 228.8 MeV. These discrete energies were carefully selected to minimize the ripples in the dose distribution along the beam direction and minimum MU effects. The energy layer switch time for all 97 energies ranged from 1.9 to 2.0 s, with an average of 1.91 s. The average spill length was 7.9 s. The average magnet preparation and verification time was 1.93 ms.^49^ The related field and energy choices, estimated delivery duration, and repainting numbers are shown in the Table S2. We used orthogonal pair kV images to align to bony anatomy during the IMPT treatment.

For both IMPT and VMAT, the set up images were reviewed offline and approved by the treating radiation oncologist to make sure that the patient setup errors were within clinical tolerance.

### 2.5 Plan quality evaluation

Due to the different treatment planning methods used for VMAT and IMPT, the same target volumes, CTV_high_ and CTV_low_, were used in plan quality, robustness, and interplay effects evaluation for fair comparison. CTV_high_ D_95%_ (the minimum dose covering the lowest 95% of the irradiated structure's volume), D_5%_, $D_{2cm^{3}}$ (the minimum dose to the highest irradiated 2cm^3^ of the structure's volume), and CTV_low_ D_95%_ were calculated from the CTV_high_ and CTV_low_ DVHs respectively. CTV D_95%_, D_5%_‐D_95%,_ and $D_{2cm^{3}}$ were used to illustrate target dose coverage, target dose homogeneity, and hot spots respectively. The CTV‐related DVH band width was normalized by the corresponding prescription dose. We evaluated OAR protection using lung D_mean_, spinal cord D_max_ and $D_{0.03cm^{3}}$, heart D_mean_, and liver D_mean_. The volumetric‐based constraints including lung V_5Gy[RBE]_ and V_20Gy[RBE]_, heart V_20Gy[RBE]_, V_30Gy[RBE]_ and V_40Gy[RBE]_, kidney V_18Gy[RBE]_, and liver V_30Gy[RBE]_ were also calculated. Additionally, the absolute volume of stomach V_45Gy[RBE]_ by cm^3^ was calculated.

### 2.6 Robustness quantification

Patient set up uncertainty is considered to be random and can be modelled as a Gaussian distribution. Range uncertainty is considered to be systematic, but range uncertainty of a large patient population can also be considered to be a Gaussian distribution.^50^ The value of 3 mm setup uncertainty and 3% range uncertainty (equal to twice the standard deviation of the setup and range uncertainty distribution) is well‐regarded in the proton therapy community. This is applicable for the treatment of distal esophageal carcinoma with plan robustness analysis and also in the use of image‐guided radiation therapy.

For IMPT plans, 13 scenarios were taken into account, including 1 nominal and 12 perturbed scenarios representing uncertainty conditions. The range uncertainty due to the CT calibration error was assumed to be ±3% of the nominal beam range, and we rigidly shifted the isocenter of the patients in the anterior–posterior (A‐P), superior–inferior (S‐I), and right–left (R‐L) directions by ±3 mm respectively. The range and isocenter shift yielded 12 perturbed scenarios.

**Figure 1:**
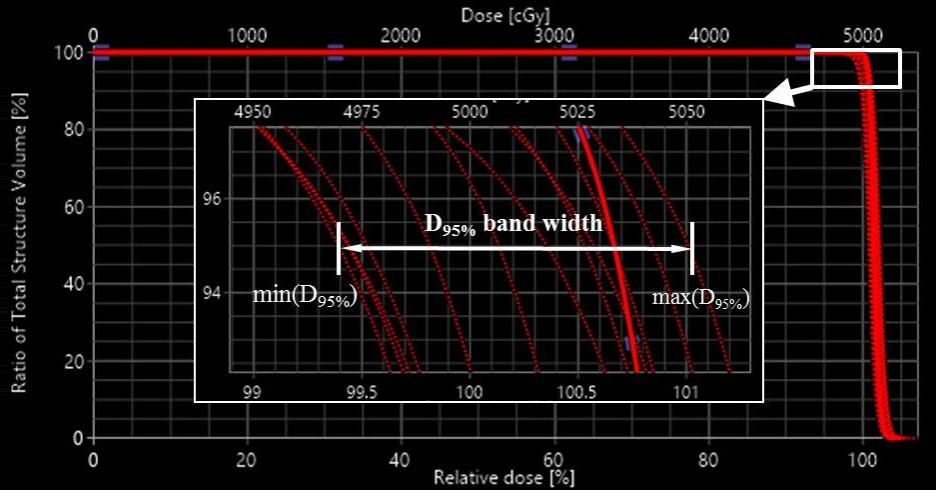
A graphical representation of the dose‐volume‐histogram band width calculating method.

For VMAT plans, seven scenarios were considered, including one nominal scenario and six perturbed scenarios. The six perturbed scenarios were created by rigidly shifting the isocenter in the same canonical directions by a distance of 3 mm, but with no range uncertainty considerations. A DVH curve was generated for each uncertainty scenario and consequently a DVH band was formed corresponding to multiple uncertainty scenarios (Fig. 1). In order to evaluate the robustness of VMAT and IMPT treatment plans, the width of the DVH band was used as a surrogate for robustness indications. The width of the DVH band was the difference between the maximum and minimum of certain DVH indices (Fig. 1). The CTV‐related DVH band width was normalized by the corresponding prescription dose. A smaller DVH band width meant better plan robustness. The DVH data for uncertainty analysis was exported from Eclipse, and the DVH band width was calculated using in‐house developed software.

All treatment plans were designed to have CTV_high_ and CTV_low_ D_95%_ reaching at least 95% of the prescription doses in the worst‐case scenarios for all CT phases.

### 2.7 Interplay effect evaluation

**Figure 2:**
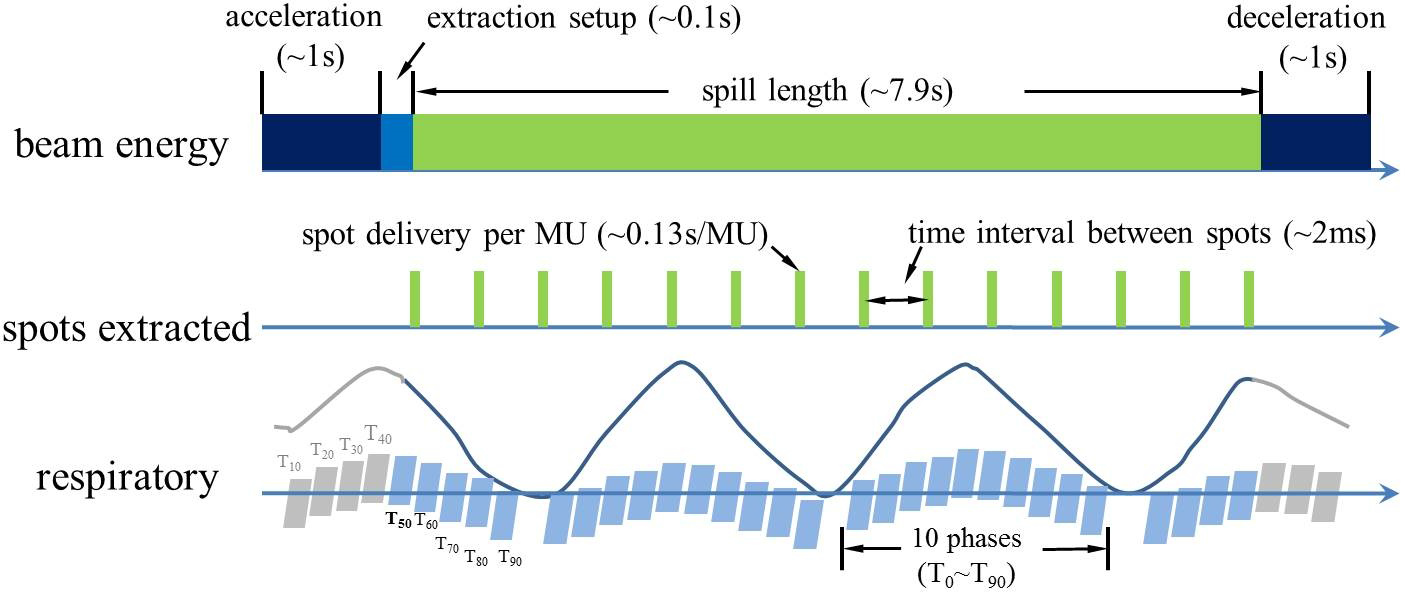
Time dependent beam spot delivery sequence and modelling of interplay effects. A random starting phase (for example T_50_) was used for each field per fraction to minimize the impact of the starting phase in the evaluation of interplay effects.

Dose evaluation software was developed to access the plans' ability to retain dose volume objectives under the influence of interplay effects. The dose evaluation software used the following machine parameters to model the time structure of spot delivery: spot delivery time per MU (i.e., dose rate), allowable extraction time (i.e., spill length), time for proton acceleration, deceleration and extraction setup, and time interval between consequent spots within the same energy layer (i.e., spot interval length). The relevant time sequence of spot delivery was shown in Fig. 2. Patient respiratory motion was modeled using 4D CTs and real‐time position management (RPM) data using the Varian RPM system (Varian Medical Systems, Palo Alto, California). Every spot of each field per fraction was assigned to the corresponding respiratory phases according to their temporal relationship with the spot delivery sequence and patient‐specific respiratory motion. The spot doses were summed to a reference phase using in‐house developed deformable image registration software.^45^, ^51^ The starting phase of each field for each fraction was randomized to minimize the influence of starting phases. The interplay effects were evaluated using the fraction number in the prescription of IMPT treatment (Table S2).

The proton absolute dose calibration process follows IAEA TRS‐398 protocol. The absolute dose of 1MU for a specific selected beam is 1 cGy. The beam used for calibration is: range 20 cm, spread out Bragg peak (SOBP) 10 cm, and field size 10 cm × 10 cm. It is composed of 27 energy layers (120–173.6 MeV), 289 spots in each layer with 6 mm spot spacing, a total of 200 MU. In IMPT treatment planning, we also used the iso‐layer repainting to mitigate interplay effects.^45^, ^51^, ^52^ For respiratory motion less than or equal to 5 mm, the minimum and maximum MU limits in the proton machine were 0.003 and 0.04 MU, respectively. A smaller maximum MU limit of 0.01 MU was used for cases with respiratory motion greater than 5 mm. The purpose of a smaller maximum MU limit was to make the delivery system perform a larger number of iso‐layer repainting, which in turn mitigated the impact of interplay effects.^52^, ^53^ During the delivery process, if the intensity of one spot was larger than the maximum MU limit, the spot was split into multiple spots, and the split spots were appended in the spot list of the same energy layer and delivered individually. For the spots with MU less than the minimum MU limit: if the intensity of one spot was larger than half of the minimum MU limit (0.0015 MU), it was rounded up; and vice versa. For example, if the amplitude of breathing motion was smaller than 5 mm, a spot of 0.121 MU was split into three spots of 0.04 MU, and the residual 0.001 MU was neglected due to its value less than half of the minimum MU limit (0.0015 MU); a spot of 0.082 MU was be split into two spots of 0.04 MU and one spot of 0.003, since the remaining 0.002 MU was larger than 0.0015 MU. If the amplitude of breathing motion was larger than 5 mm, a spot of 0.121 MU was split into 12 spots of 0.01 MU, and the residual 0.001 MU was neglected due to its value less than half of the minimum MU limit (0.0015 MU); a spot of 0.082 MU was split into eight spots of 0.01 MU and one spot of 0.003, since the remaining 0.002 MU was larger than 0.0015 MU. No interplay effects were evaluated for VMAT planning, as it has been shown that the impact was small and not clinically significant.^54^, ^55^

### 2.8 Statistical analysis

For fair comparison, all the treatment plans were normalized to have the same CTV_high_ D_95%_ in the nominal scenario relative to the prescription dose. Wilcoxon Rank‐sum and Fisher's exact test were utilized to compare clinical characteristics between treatment populations for continuous and categorical data respectively. *P* < 0.003 (0.05/16 = 0.003) were considered statistically significant after adjusting for 16 different DVH indices. The results of the DVH indices from all patients were presented using Box‐and‐Whisker plotting which showed median values along with error bars. The maximum and minimum outlier points were located outside of the error bars which corresponded to 1.5 times the interquartile range above the upper and below the lower quartiles.

## 3 RESULTS

### 3.1 Plan quality

**Figure 3:**
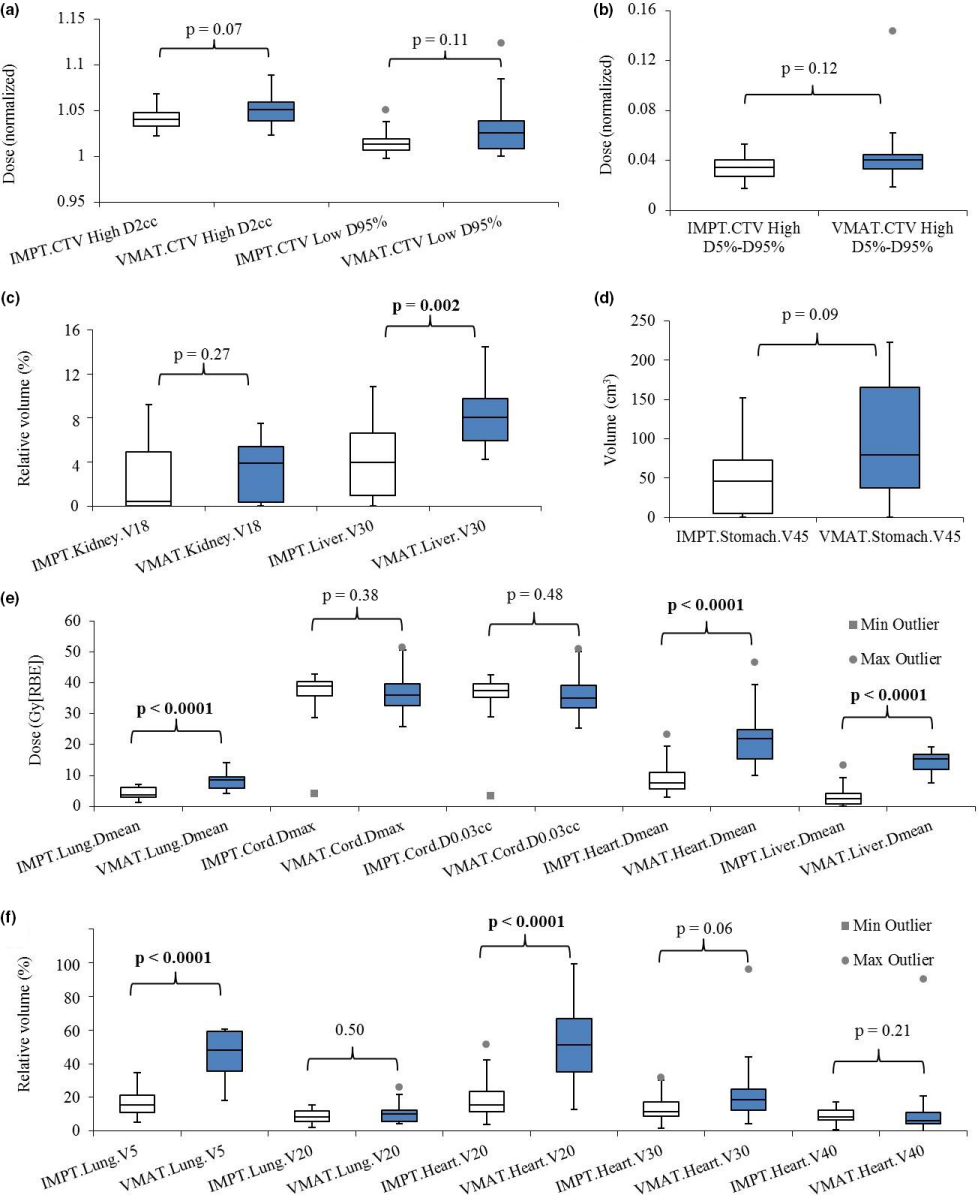
Comparison of the dose‐volume‐histogram (DVH) indices between intensity‐modulated proton therapy (IMPT) and volumetric‐modulated arc therapy (VMAT) treatment plans in the nominal scenario. (a) clinical target volume (CTV_high_) $D_{2cm^{3}}$ and CTV_low_ D_95%_ normalized to the prescription dose. (b) CTV_high_ D_5%_–D_95%_ normalized to the prescription dose. (c) Kidney V_18Gy[RBE]_ and liver V_30Gy[RBE]_ in relative volume. (d) Absolute volume stomach V_45Gy[RBE]_ in absolute volume. (e) Lung D_mean_, cord D_max_ and $D_{0.03cm^{3}}$_,_ heart D_mean_, and liver D_mean_ in absolute dose. (f) Lung V_5Gy[RBE]_ and V_20Gy[RBE]_, heart V_20Gy[RBE]_, V_30Gy[RBE]_ and V_40Gy[RBE]_ in relative volume. In each box plot, the five horizontal lines from top to bottom are the maximum, third quartile, median, first quartile, and minimum value of the corresponding DVH index for the whole group excluding the outliers respectively. The grey points are the outliers as defined in Statistical Analysis subsection. Numbers at the top of the columns are *P*‐values from Wilcoxon rank sum testing. The blue boxes are the IMPT results and white boxes are the VMAT results. Abbreviations: RBE = relative biological effectiveness.

Considering plan quality, we first compared the DVH indices of IMPT and VMAT in the nominal scenario (without any uncertainties considered). The IMPT plans performed significantly better in terms of liver V_30Gy[RBE]_, lung D_mean_ and V_5Gy[RBE]_, heart D_mean_ and V_20Gy[RBE]_, and liver D_mean_ [Figs. 3(c)–3(f); Table 3]. Compared to the VMAT plans, IMPT plans did not significantly differ with respect to the following: CTV_high_ $D_{2cm^{3}}$ (normalized by the prescription doses), CTV_high_ D_5%_–D_95%_ (normalized by the prescription doses), CTV_low_ dose coverage, and protection of other OARs including kidney V_18Gy[RBE]_, stomach V_45Gy[RBE]_, cord D_max_ and $D_{0.03cm^{3}}$, lung V_20Gy[RBE]_, and heart V_30Gy[RBE]_ and V_40Gy[RBE]_ [Figs. 3(a)–3(f); Table 3].

**Table 3:** The comparison of plan quality using dose‐volume‐histogram (DVH) indices.

DVH index	VMAT	IMPT	*P*‐value
CTV_high_ ${\text{D}}_{2{\text{cm}}^{3}}$ (normalized)	105%	104%	0.071
CTV_high_ D_5%_–D_95%_ (normalized)	4.0%	3.5%	0.12
CTV_low_ D_95%_ (normalized)	103%	101%	0.11
Liver V_30Gy[RBE]_ (%)	8.05%	**3.97%**	**0.0023**
Liver D_mean_ (Gy[RBE])	15.27	**2.58**	**<0.0001**
Lung D_mean_ (Gy[RBE])	8.55	**3.73**	**<0.0001**
Lung V_5Gy[RBE]_ (%)	48.11%	**15.61%**	**<0.0001**
Lung V_20Gy[RBE]_ (%)	10.32%	8.57%	0.50
Heart D_mean_ (Gy[RBE])	21.94	**7.63**	**<0.0001**
Heart V_20Gy[RBE]_ (%)	51.06%	**15.61%**	**<0.0001**
Heart V_30Gy[RBE]_ (%)	18.52%	11.48%	0.061
Heart V_40Gy[RBE]_ (%)	8.38%	6.31%	0.21
Cord D_max_ (Gy[RBE])	36.00	38.92	0.38
Cord ${\text{D}}_{0.03{\text{cm}}^{3}}$ (Gy[RBE])	35.05	37.49	0.48
Kidney V_18Gy[RBE]_ (%)	3.88%	0.41%	0.27
Stomach V_45Gy[RBE]_ (cm^3^)	79.92	45.71	0.093

CTV, clinical target volume; IMPT, intensity‐modulated proton therapy; VMAT, volumetric‐modulated arc therapy.

Bold values represent significant difference between IMPT and VMAT DVH indices.

Some outliers were observed. For example, heart D_mean_, V_20Gy[RBE]_, and V_30Gy[RBE]_ in IMPT [Figs. 3(e)–3(f)]. These outliers were found to come from the same patient. The heart was in close proximity to the CTV_high_ [Fig. S1(a)], which resulted in higher dose to the heart [Fig. S1(b)]. Similarly, close proximity of the heart to a tumor resulted in the outliers in heart V_30Gy[RBE]_ and V_40Gy[RBE]_ [Fig. 3(f)] in VMAT. To avoid vital organs including cord and lungs for this patient, we had to limit target dose coverage and homogeneity to some degree in VMAT, resulting in additional outliers in D_5%_–D_95%_ [Fig. 3(b)].

### 3.2 Plan robustness

**Figure 4:**
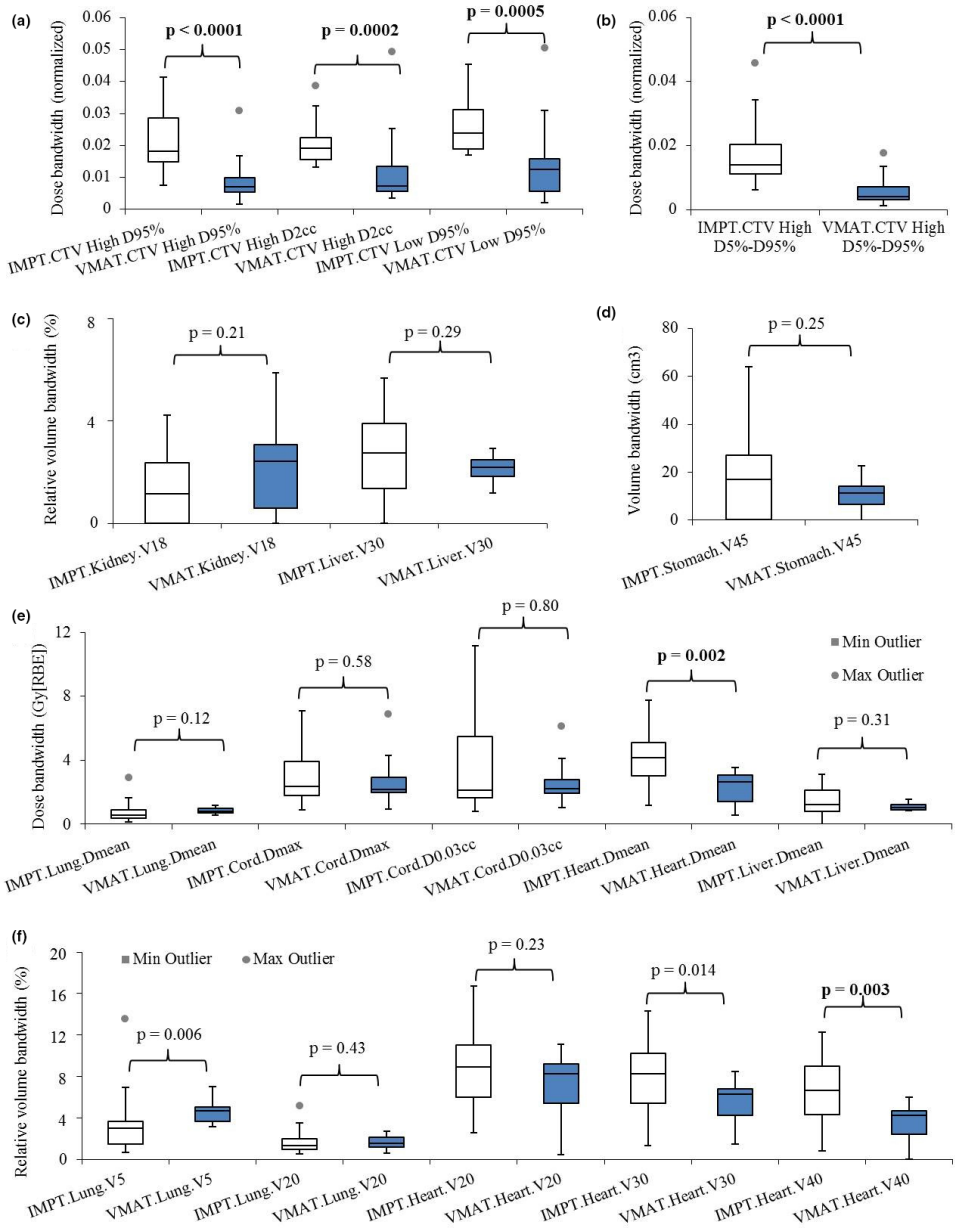
Comparison of plan robustness between intensity‐modulated proton therapy (IMPT) and volumetric‐modulated arc therapy (VMAT) plans using the widths of the dose volume histogram (DVH) band pairs in the presence of uncertainties. (a) clinical target volume (CTV_high_) D_95%_ and $D_{2cm^{3}}$, and CTV_low_ D_95%_ normalized to the prescription dose. (b) CTV_high_ D_5%_‐D_95%_ normalized to the prescription dose. (c) Kidney V_18Gy[RBE]_ and liver V_30Gy[RBE]_ in relative volume. (d) Stomach V_45Gy[RBE]_ in absolute volume. (e) Lung D_mean_, cord D_max_ and $D_{0.03cm^{3}}$_,_ heart D_mean_, and liver D_mean_ in absolute dose. (f) Lung V_5Gy[RBE]_ and V_20Gy[RBE]_, heart V_20Gy[RBE]_, V_30Gy[RBE]_ and V_40Gy[RBE]_ in relative volume. In each box plot, the five horizontal lines from top to bottom are the maximum, third quartile, median, first quartile, and minimum value of the DVH band widths in the presence of uncertainties of the corresponding DVH index for the whole group excluding the outliers respectively. The grey points are the outliers as defined in Statistical Analysis subsection. Numbers at the top of the columns are *P*‐values from Wilcoxon rank sum testing. The blue boxes are the IMPT results and white boxes are the VMAT results. Abbreviations: RBE = relative biological effectiveness.

Considering the plan robustness, the DVH index range of CTVs and OARs for both treatment groups were compared (Table 4). The robustness of IMPT plans was comparable to that of VMAT plans for kidney V_18Gy[RBE]_, liver V_30Gy[RBE]_, stomach V_45Gy[RBE]_, lung D_mean_, V_5Gy[RBE]_ and V_20Gy[RBE]_, cord D_max_ and $D_{0.03cm^{3}}$, liver D_mean_, heart V_20Gy[RBE]_ and V_30Gy[RBE]_. The robustness of IMPT plans was statistically worse than that of VMAT plans for CTV_high_ D_95%_ (normalized by the prescription doses), CTV_high_ $D_{2cm^{3}}$ (normalized by the prescription doses), CTV_high_ D_5%_–D_95%_ (normalized by the prescription doses), CTV_low_ D_95%_ (normalized by the prescription doses), heart D_mean_ and V_40Gy[RBE]_ (Fig. 4).

**Table 4:** The comparison of plan robustness using the width of dose‐volume‐histogram (DVH) index bands.

DVH index	VMAT	IMPT	*P*‐value
CTV_high_ D_95%_ (normalized)	**0.7%**	1.8%	**<0.0001**
CTV_high_ ${\text{D}}_{2{\text{cm}}^{3}}$ (normalized)	**0.73%**	1.9%	**0.0002**
CTV_high_ D_5%_–D_95%_ (normalized)	**0.41%**	1.4%	**<0.0001**
CTV_low_ D_95%_ (normalized)	**1.3%**	2.4%	**0.0005**
Liver V_30Gy[RBE]_ (%)	2.19%	2.76%	0.29
Liver D_mean_ (Gy[RBE])	1.03	1.22	0.31
Lung D_mean_ (Gy[RBE])	0.80	0.58	0.12
Lung V_5Gy[RBE]_ (%)	4.73%	2.99%	0.0057
Lung V_20Gy[RBE]_ (%)	1.58%	1.31%	0.43
Heart D_mean _(Gy[RBE])	**2.65**	4.13	**0.002**
Heart V_20Gy[RBE]_ (%)	8.28%	8.91%	0.23
Heart V_30Gy[RBE]_ (%)	**6.31%**	8.24%	**0.014**
Heart V_40Gy[RBE]_ (%)	**4.28%**	6.69%	**0.0027**
Cord D_max_ (Gy[RBE])	2.17	2.35	0.58
Cord ${\text{D}}_{0.03{\text{cm}}^{3}}$ (Gy[RBE])	2.21	2.12	0.80
Kidney V_18Gy[RBE]_ (%)	2.42%	1.16%	0.21
Stomach V_45Gy[RBE]_ (cm^3^)	11.34	16.99	0.25

CTV, clinical target volume; IMPT, intensity‐modulated proton therapy; VMAT, volumetric‐modulated arc therapy.

Bold values represent significant difference between IMPT and VMAT DVH indices.

### 3.3 Interplay effect

**Figure 5:**
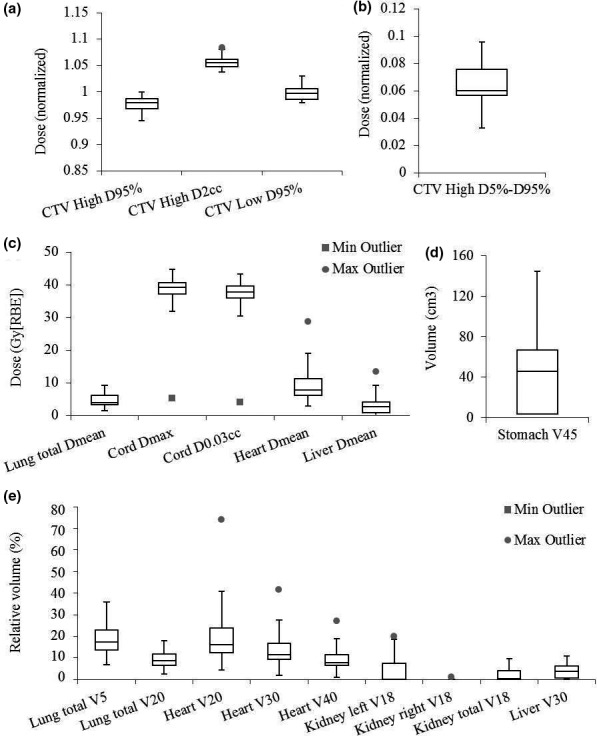
Interplay effects evaluation in intensity‐modulated proton therapy (IMPT) using the dose–volume histogram indices with interplay effects considered, including (a) clinical target volume (CTV_high_) D_95%_ and$D_{2cm^{3}}$, and CTV_low_ D_95%_ normalized to the prescription dose. (b) CTV_high_ D_5%_‐D_95%_ normalized to the prescription dose. (c) Lung D_mean_, cord D_max_ and$D_{0.03cm^{3}}$_,_ heart D_mean_, and liver D_mean_ in absolute dose. (d) Stomach V_45Gy[RBE]_ in absolute volume. (e) Lung V_5Gy[RBE]_ and V_20Gy[RBE]_, heart V_20Gy[RBE]_, V_30Gy[RBE]_ and V_40Gy[RBE]_, left, right, and total kidneys V_18Gy[RBE]_, and liver V_30Gy[RBE]_ in relative volume. In each box plot, the five horizontal lines from top to bottom are the maximum, third quartile, median, first quartile, and minimum value of the corresponding DVH index for the whole group excluding the outliers respectively. The grey points are the outliers as defined in Statistical Analysis subsection. The blue boxes are the IMPT results and white boxes are the VMAT results. At our clinics it is required to have CTV D_95%_ is at least 95% of the prescription dose for targets and the cord D_max_ < 45 Gy[RBE] under the influence of interplay effects. Abbreviations: RBE = relative biological effectiveness.

Interplay effects were considered for all of the IMPT plans [see Fig. 5(a)–5(d)]. The median values of CTV_high_ D_95%_, $D_{2cm^{3}}$, and D_5%_–D_95%_ (normalized by the prescription doses) were 0.98, 1.06, and 0.06, respectively. The median value of CTV_low_ D_95%_ was 0.99. Median values of total lung V_5Gy[RBE]_ and V_20Gy[RBE]_, heart V_20Gy[RBE]_, V_30Gy[RBE]_ and V_40Gy[RBE]_, liver V_30Gy[RBE]_, left, right and total kidney V_18Gy[RBE]_ were 17.4%, 8.7%, 16.1%, 11.6%, 7.7%, 3.8%, 0%, 0%, and 0.09% respectively. Median values of total lung D_mean_, spinal cord D_max_ and $D_{0.03cm^{3}}$, heart D_mean_, liver D_mean_ were 4.0 Gy[RBE], 39.2 Gy[RBE], 37.8 Gy[RBE], 7.8 Gy[RBE], 2.7 Gy[RBE] respectively.

## 4 DISCUSSION

The purpose of this comparative planning study was to evaluate the process and feasibility of small‐spot machine IMPT in the treatment for distal esophagus carcinoma as well as provide a dosimetric comparison to VMAT in regard to plan quality and robustness.

Small spot‐size IMPT is an attractive modality for the treatment of esophageal cancer. Our study compared IMPT with spot sizes of 2–6 mm (σ) with VMAT. We found that small‐spot IMPT achieved similar plan quality as VMAT in terms of target dose coverage, homogeneity, and sparing of most OARs. More importantly, it significantly lowered heart, liver, and bilateral lung doses as compared to VMAT. As a result, IMPT will likely reduce the incidence and also severity of RT‐induced cardiac and pulmonary toxicities in the long‐term and perioperatively. However, the equipoise of such considerations and clinically meaningful significance over conventional treatments such as IMRT or VMAT remain undefined for distal esophageal cancer therapies.

Although small‐spot IMPT is potentially capable of producing better plans due to sharper penumbra, the uncertainties due to proton range and patient setup can greatly compromise plan qualities.^56^, ^57^ Therefore, it is important to take into account the uncertainties in treatment planning when using a small‐spot IMPT machine to treat patients with esophageal carcinoma. Our study demonstrates that small‐spot IMPT treatment plans achieved clinically defined planning requirements in terms of plan robustness and met the clinical standards for RT. Small‐spot IMPT should be considered for the routine treatment in patients with distal esophageal carcinoma. However, robustness relative to internal organ motion remains a major challenge in small‐spot IMPT treatments for esophageal cance.^30^, ^33^, ^45^, ^53^ Compared with IMPT, VMAT has been shown to be more robust with respect to organ motions or anatomic changes.^58^ Therefore, it is vital to consider and optimally mitigate the impact of respiratory motions when IMPT is clinically implemented. At our institution, we carefully consider respiratory motion and its impact in our treatment planning guidelines. Compared with IMRT, large‐spot IMPT using three beam angles (anteroposterior, right posterior oblique, and left posterior oblique) has been shown to deliver significantly lower lung D_mean_, lung V_5Gy[RBE]_, V_10Gy[RBE]_, and V_20Gy[RBE]_, and heart D_mean_ for distal esophageal cancer patients.^37^ Compared with large‐spot IMPT, small‐spot IMPT is considered to have two benefits: (a) sharper penumbra, which results from smaller spot sizes, can improve the sparing of organs‐at‐risk and lead to lower radiation toxicities^52^, ^59^; (b) a larger number of spots, which are needed to cover the same tumors compared to large‐spot IMPT, provides the TPS more freedom to compensate for the impact of uncertainties and interplay effects.^53^

Most of the IMPT plans included in this study were generated using a SFO approach. The few remaining plans were generated using MFO with robust planning from our TPS.^39^, ^40^, ^41^, ^42^, ^43^ After the plan was optimized, we recalculated the dose distributions in the averaged 4D CT, maximum exhale phase CT, and maximum inhale phase CT without density override to evaluate the impact of respiratory motion. The original plan generated on the averaged CT was adjusted until all dose distributions calculated on the averaged CT, maximum exhale phase CT, and maximum inhale phase CT without density override met the institutional dose constraints. All patients were observed to have both CTV_high_ and CTV_low_ D_95%_ at least 95% of the prescription dose with interplay effects taken into account. As a result, even though some of the patients included in this study have considerable amount of target motion as influenced by respiration (the median value of the amplitude of respiratory motions was 8 mm, with a range of 4 to 13 mm), all of our plans using small‐spot IMPT still met the clinical requirement in terms of robustness and interplay effect considerations after these treatment planning measures were taken.

For distal esophageal carcinoma, we commonly used posterior oblique beams in our IMPT treatment: left–right oblique beams or superior‐inferior oblique beams. Left–right oblique beams spare the spinal cord, but may be sensitive to respiratory motion (Fig. S2). Superior‐inferior oblique beams spare the kidneys and have better plan robustness to respiratory motion since the beam does not travel through much of the diaphragm. The above beam angle arrangements worked very well for most patients. However, in some scenarios, there were variants of beam arrangements for specific patients: if the patient was pretreated by radiation therapy and the dose in certain organs needed to be limited, three or four beams were used to spare certain organs (Table S2); if the patient had significant respiratory motion and two beams could not achieve the plan robustness of clinical requirements, then three or four beams were used to improve the plan robustness (Table S2).

This study has a number of limitations. First, the number of the patients included in this study was not sufficiently large nor were they matched. The results could be affected by interpatient variability and different planning skills. However, these represented consecutive samples of actual IMPT vs VMAT plans that were delivered in the clinic over a similar period of time. To address the aforementioned issues, a study with a larger patient population with both VMAT and IMPT plans generated for each patient is currently under way to further generalize our conclusions. Second, only a limited number of uncertainty scenarios were considered in this study, which might underestimate the impact of uncertainties in selected IMPT plans.

In the future, other tumor locations (cervical, proximal, and mid‐esophageal) should be considered in further studies. Furthermore, more patient data with short/long‐term clinical outcomes, perioperative complications, gradation‐related toxicities, and patient‐reported outcome should be reported to evaluate the potential clinical benefits of small‐spot IMPT over VMAT plans.

## 5 CONCLUSION

Compared to VMAT, small‐spot IMPT significantly improves RT sparing of the heart, liver, and lungs, as well as achieves clinically acceptable plan robustness. The impact of interplay effects is small when proper treatment planning and respiratory motion measures are taken. Our results support the feasibility and acceptability for the routine clinical use of small‐spot IMPT in patients with distal esophageal carcinoma.

## CONFLICTS OF INTEREST

None.

## ETHICAL CONSIDERATIONS

This research was approved by the Mayo Clinic Arizona institutional review board (IRB, 13‐005709). The informed consent was waived by IRB protocol. Only image and dose‐volume data were used in this study. All patient‐related health information was removed for the study.

## References

[1] Herszenyi L , Tulassay Z . Epidemiology of gastrointestinal and liver tumors. Eur Rev Med Pharmacol Sci. 2010;14:249–258. 20496531

[2] Torre LA , Bray F , Siegel RL , Ferlay J , Lortet‐Tieulent J , Jemal A . Global cancer statistics, 2012. CA. 2015;65:87–108. 25651787 10.3322/caac.21262

[3] van Hagen P , Hulshof M , Van Lanschot J , et al. Preoperative chemoradiotherapy for esophageal or junctional cancer. N Engl J Med. 2012;366:2074–2084. 22646630 10.1056/NEJMoa1112088

[4] Berger B , Belka C . Evidence‐based radiation oncology: oesophagus. Radiother Oncol. 2009;92:276–290. 19375187 10.1016/j.radonc.2009.02.019

[5] Cooper JS , Guo MD , Herskovic A , et al. Chemoradiotherapy of locally advanced esophageal cancer: long‐term follow‐up of a prospective randomized trial (RTOG 85–01). JAMA. 1999;281:1623–1627. 10235156 10.1001/jama.281.17.1623

[6] Gharzai L , Verma V , Denniston KA , Bhirud AR , Bennion NR , Lin C . Radiation therapy and cardiac death in long‐term survivors of esophageal cancer: an analysis of the Surveillance, Epidemiology, and End Result database. PLoS ONE. 2016;11:e0158916. 27428362 10.1371/journal.pone.0158916 PMC4948887

[7] Kole TP , Aghayere O , Kwah J , Yorke ED , Goodman KA . Comparison of heart and coronary artery doses associated with intensity‐modulated radiotherapy versus three‐dimensional conformal radiotherapy for distal esophageal cancer. Int J Radiat Oncol Biol Phys. 2012;83:1580–1586. 22284687 10.1016/j.ijrobp.2011.10.053

[8] Liu W , Patel SH , Shen JJ , et al. Robustness quantification methods comparison in volumetric modulated arc therapy to treat head and neck cancer. Pract Radiat Oncol. 2016;6:e269–e275. 27025166 10.1016/j.prro.2016.02.002 PMC4983261

[9] Van Benthuysen L , Hales L , Podgorsak MB . Volumetric modulated arc therapy vs. IMRT for the treatment of distal esophageal cancer. Med Dosim. 2012;36:404–409. 10.1016/j.meddos.2010.09.009 21377864

[10] Gong G , Wang R , Guo Y , et al. Reduced lung dose during radiotherapy for thoracic esophageal carcinoma: VMAT combined with active breathing control for moderate DIBH. Radiat Oncol. 2013;8:291. 24359800 10.1186/1748-717X-8-291 PMC3896728

[11] Deasy JO , Zuniga A , Apte A , Thorstad W , Bradley J . A new tumor control probability model fitted to head‐and‐neck and lung local control data and resulting implications for the impact of dose heterogeneity. Int J Radiat Oncol Biol Phys. 2013;87:S694.

[12] Register SP , Zhang X , Mohan R , Chang JY . Proton stereotactic body radiation therapy for clinically challenging cases of centrally and superiorly located stage I non‐small‐cell lung cancer. Int J Radiat Oncol Biol Phys. 2011;80:1015–1022. 20615629 10.1016/j.ijrobp.2010.03.012 PMC2952351

[13] Zhang X , Li Y , Pan X , et al. Intensity‐modulated proton therapy reduces the dose to normal tissue compared with intensity‐modulated radiation therapy or passive scattering proton therapy and enables individualized radical radiotherapy for extensive stage IIIB non‐small‐cell lung cancer: a virtual clinical study. Int J Radiat Oncol Biol Phys. 2010;77:357–366. 19660879 10.1016/j.ijrobp.2009.04.028 PMC2868090

[14] Sejpal S , Komaki R , Tsao A , et al. Early findings on toxicity of proton beam therapy with concurrent chemotherapy for nonsmall cell lung cancer. Cancer. 2011;117:3004–3013. 21264827 10.1002/cncr.25848

[15] Chang JY , Li H , Zhu XR , et al. Clinical implementation of intensity modulated proton therapy for thoracic malignancies. Int J Radiat Oncol Biol Phys. 2014;90:809–818. 25260491 10.1016/j.ijrobp.2014.07.045 PMC4252731

[16] Berman AT , Teo B‐KK , Dolney D , et al. An in‐silico comparison of proton beam and IMRT for postoperative radiotherapy in completely resected stage IIIA non‐small cell lung cancer. Radiat Oncol. 2013;8:144. 23767810 10.1186/1748-717X-8-144 PMC3695889

[17] Schneider U , Pedroni E , Lomax A . The calibration of CT Hounsfield units for radiotherapy treatment planning. Phys Med Biol. 1996;41:111–124. 8685250 10.1088/0031-9155/41/1/009

[18] Kraus KM , Heath E , Oelfke U . Dosimetric consequences of tumor motion due to respiration for a scanned proton beam. Phys Med Biol. 2011;56:6563–6581. 21937770 10.1088/0031-9155/56/20/003

[19] Phillips MH , Pedroni E , Blattmann H , Boehringer T , Coray A , Scheib S . Effects of respiratory motion on dose uniformity with a charged‐particle scanning method. Phys Med Biol. 1992;37:223–234. 1311106 10.1088/0031-9155/37/1/016

[20] Lambert J , Suchowerska N , McKenzie DR , Jackson M . Intrafractional motion during proton beam scanning. Phys Med Biol. 2005;50:4853–4862. 16204877 10.1088/0031-9155/50/20/008

[21] Grozinger SO , Bert C , Haberer T , Kraft G , Rietzel E . Motion compensation with a scanned ion beam: a technical feasibility study. Radiat Oncol. 2008;3:34. 18854012 10.1186/1748-717X-3-34 PMC2576303

[22] Seco J , Robertson D , Trofimov A , Paganetti H . Breathing interplay effects during proton beam scanning: simulation and statistical analysis. Phys Med Biol. 2009;54:N283–N294. 19550002 10.1088/0031-9155/54/14/N01

[23] Grozinger SO , Rietzel E , Li Q , Bert C , Haberer T , Kraft G . Simulations to design an online motion compensation system for scanned particle beams. Phys Med Biol. 2006;51:3517–3531. 16825746 10.1088/0031-9155/51/14/016

[24] Dowdell S , Grassberger C , Sharp GC , Paganetti H . Interplay effects in proton scanning for lung: a 4D Monte Carlo study assessing the impact of tumor and beam delivery parameters. Phys Med Biol. 2013;58:4137–4156. 23689035 10.1088/0031-9155/58/12/4137 PMC3752993

[25] Grassberger C , Dowdell S , Lomax A , et al. Motion interplay as a function of patient parameters and spot size in spot scanning proton therapy for lung cancer. Int J Radiat Oncol Biol Phys. 2013;86:380–386. 23462423 10.1016/j.ijrobp.2013.01.024 PMC3646997

[26] Knopf A‐C , Hong TS , Lomax A . Scanned proton radiotherapy for mobile targets‐the effectiveness of re‐scanning in the context of different treatment planning approaches and for different motion characteristics. Phys Med Biol. 2011;56:7257–7271. 22037710 10.1088/0031-9155/56/22/016

[27] Li Y , Kardar L , Li X , et al. On the interplay effects with proton scanning beams in stage III lung cancer. Med Phys. 2014;41:021721. 24506612 10.1118/1.4862076 PMC4108709

[28] Kardar L , Li Y , Li X , et al. Evaluation and mitigation of the interplay effects of intensity modulated proton therapy for lung cancer in a clinical setting. Pract Radiat Oncol. 2014;4:e259–e268. 25407877 10.1016/j.prro.2014.06.010 PMC4399385

[29] Bortfeld T , Jokivarsi K , Goitein M , Kung J , Jiang SB . Effects of intra‐fraction motion on IMRT dose delivery: statistical analysis and simulation. Phys Med Biol. 2002;47:2203–2220. 12164582 10.1088/0031-9155/47/13/302

[30] Liu W , Schild SE , Chang JY , et al. Exploratory study of 4D versus 3D robust optimization in intensity modulated proton therapy for lung cancer. Int J Radiat Oncol Biol Phys. 2016;95:523–533. 26725727 10.1016/j.ijrobp.2015.11.002 PMC4834263

[31] Moteabbed M , Yock TI , Depauw N , Madden TM , Kooy HM , Paganetti H . Impact of spot size and beam‐shaping devices on the treatment plan quality for pencil beam scanning proton therapy. Int J Radiat Oncol Biol Phys. 2016;95:190–198. 27084640 10.1016/j.ijrobp.2015.12.368 PMC4834139

[32] Shiraishi Y , Xu C , Yang J , Komaki R , Lin SH . Dosimetric comparison to the heart and cardiac substructure in a large cohort of esophageal cancer patients treated with proton beam therapy or Intensity‐modulated radiation therapy. Radiother Oncol. 2017;125:48–54. 28917586 10.1016/j.radonc.2017.07.034

[33] Chuong MD , Hallemeier CL , Jabbour SK , et al. Improving outcomes for esophageal cancer using proton beam therapy. Int J Radiat Oncol Biol Phys. 2016;95:488–497. 27084662 10.1016/j.ijrobp.2015.11.043 PMC10862360

[34] Lin SH , Komaki R , Liao Z , et al. Proton beam therapy and concurrent chemotherapy for esophageal cancer. Int J Radiat Oncol Biol Phys. 2012;83:e345–e351. 22417808 10.1016/j.ijrobp.2012.01.003 PMC3923631

[35] Zhang X , Guerrero TM , Mcguire SE , et al. Four‐dimensional computed tomography–based treatment planning for intensity‐modulated radiation therapy and proton therapy for distal esophageal cancer. Int J Radiat Oncol Biol Phys. 2008;72:278–287. 18722278 10.1016/j.ijrobp.2008.05.014 PMC2610812

[36] Lin C‐Y , Huang W‐Y , Jen Y‐M , et al. Dosimetric and efficiency comparison of high‐dose radiotherapy for esophageal cancer: volumetric modulated arc therapy versus fixed‐field intensity‐modulated radiotherapy. Dis Esophagus. 2014;27:585–590. 24134466 10.1111/dote.12144

[37] Welsh J , Gomez D , Palmer MB , et al. Intensity‐modulated proton therapy further reduces normal tissue exposure during definitive therapy for locally advanced distal esophageal tumors: a dosimetric study. Int J Radiat Oncol Biol Phys. 2011;81:1336–1342. 21470796 10.1016/j.ijrobp.2010.07.2001 PMC4086056

[38] Lin SH , Merrell KW , Shen J , et al. Multi‐institutional analysis of radiation modality use and postoperative outcomes of neoadjuvant chemoradiation for esophageal cancer. Radiother Oncol. 2017;123:376–381. 28455153 10.1016/j.radonc.2017.04.013

[39] Liu W , Frank SJ , Li X , et al. Effectiveness of robust optimization in intensity‐modulated proton therapy planning for head and neck cancers. Med Phys. 2013;40:051711–051718. 23635259 10.1118/1.4801899 PMC3651255

[40] Liu W , Frank SJ , Li X , Li Y , Zhu RX , Mohan R . PTV‐based IMPT optimization incorporating planning risk volumes vs robust optimization. Med Phys. 2013;40:021709–021708. 23387732 10.1118/1.4774363 PMC3562272

[41] Liu W , Li Y , Li X , Cao W , Zhang X . Influence of robust optimization in intensity‐modulated proton therapy with different dose delivery techniques. Med Phys. 2012;39:3089–3101. 22755694 10.1118/1.4711909 PMC3360691

[42] Liu W , Mohan R , Park P , et al. Dosimetric benefits of robust treatment planning for intensity modulated proton therapy for base‐of‐skull cancers. Pract Radiat Oncol. 2014;4:384–391. 25407859 10.1016/j.prro.2013.12.001 PMC4238033

[43] Liu W , Zhang X , Li Y , Mohan R . Robust optimization in intensity‐modulated proton therapy. Med Phys. 2012;39:1079–1091. 22320818 10.1118/1.3679340 PMC3281975

[44] Kang Y , Zhang X , Chang JY , et al. 4D proton treatment planning strategy for mobile lung tumors. Int J Radiat Oncol Biol Phys. 2007;67:906–914. 17293240 10.1016/j.ijrobp.2006.10.045

[45] Liu W , Liao Z , Schild SE , et al. Impact of respiratory motion on worst‐case scenario optimized intensity modulated proton therapy for lung cancers. Pract Radiat Oncol. 2015;5:e77–e86. 25413400 10.1016/j.prro.2014.08.002 PMC4355168

[46] Wan Chan Tseung H , Ma J , Beltran C . A fast GPU‐based Monte Carlo simulation of proton transport with detailed modeling of nonelastic interactions. Med Phys. 2015;42:2967–2978. 26127050 10.1118/1.4921046

[47] Shen J , Liu W , Stoker J , et al. An efficient method to determine double Gaussian fluence parameters in the eclipse™ proton pencil beam model. Med Phys. 2016;43:6544–6551. 27908162 10.1118/1.4967485 PMC6961730

[48] Shen J , Lentz JM , Hu Y , et al. Using field size factors to characterize the in‐air fluence of a proton machine with a range shifter. Radiat Oncol. 2017;12:52. 28288673 10.1186/s13014-017-0783-2 PMC5348744

[49] Shen J , Tryggestad E , Younkin JE , et al. Using experimentally determined proton spot scanning timing parameters to accurately model beam delivery time. Med Phys. 2017;44:5081–5088. 28777447 10.1002/mp.12504

[50] Pflugfelder D , Wilkens JJ , Oelfke U . Worst case optimization: a method to account for uncertainties in the optimization of intensity modulated proton therapy. Phys Med Biol. 2008;53:1689–1700. 18367797 10.1088/0031-9155/53/6/013

[51] Liu W , Schild S , Chang J , et al. A novel 4D robust optimization mitigates interplay effect in intensity‐modulated proton therapy for lung cancer. Med Phys. 2015;42:3525.

[52] Liu C , Sio TT , Deng W , et al. Small‐spot intensity‐modulated proton therapy and volumetric‐modulated arc therapies for patients with locally advanced non‐small‐cell lung cancer: a dosimetric comparative study. J Appl Clin Med Phys. 2018;19:140–148. 30328674 10.1002/acm2.12459 PMC6236833

[53] Liu C , Schild SE , Chang JY , et al. Impact of spot size and spacing on the quality of robustly optimized intensity modulated proton therapy plans for lung cancer. Int J Radiat Oncol Biol Phys. 2018;101:479–489. 29550033 10.1016/j.ijrobp.2018.02.009 PMC5935576

[54] Hawkins M , Bedford J , Warrington A , Tait D . Volumetric modulated arc therapy planning for distal oesophageal malignancies. Br J Radiol. 2012;85:44–52. 21427179 10.1259/bjr/25428720 PMC3473937

[55] Nicolini G , Ghosh‐Laskar S , Shrivastava SK , et al. Volumetric modulation arc radiotherapy with flattening filter‐free beams compared with static gantry IMRT and 3D conformal radiotherapy for advanced esophageal cancer: a feasibility study. Int J Radiat Oncol Biol Phys. 2012;84:553–560. 22386376 10.1016/j.ijrobp.2011.12.041

[56] Lomax AJ . Intensity modulated proton therapy and its sensitivity to treatment uncertainties 1: the potential effects of calculational uncertainties. Phys Med Biol. 2008;53:1027–1042. 18263956 10.1088/0031-9155/53/4/014

[57] Lomax AJ . Intensity modulated proton therapy and its sensitivity to treatment uncertainties 2: the potential effects of inter‐fraction and inter‐field motions. Phys Med Biol. 2008;53:1043–1056. 18263957 10.1088/0031-9155/53/4/015

[58] Chang JY , Jabbour SK , De Ruysscher D , et al. Consensus statement on proton therapy in early‐stage and locally advanced non‐small cell lung cancer. Int J Radiat Oncol Biol Phys. 2016;95:505–516. 27084663 10.1016/j.ijrobp.2016.01.036 PMC10868643

[59] Yu NY , DeWees TA , Liu C , et al. Early outcomes of patients with locally advanced non‐small cell lung cancer treated with intensity‐modulated proton therapy (IMPT) vs. intensity‐modulated radiation therapy (IMRT): a single‐institutional experience. Adv Radiat Oncol. 2019;104:234–235. 10.1016/j.adro.2019.08.001 PMC7276663 32529140

