# Supplementary material for: Dosimetric comparison of distal esophageal carcinoma plans for patients treated with small‐spot intensity‐modulated proton versus volumetric‐modulated arc therapies
Source: J Appl Clin Med Phys. 2019 May 21;20(7):15–27. doi: 10.1002/acm2.12623 (PMC6612702; doi:10.1002/acm2.12623)
Supplement: Supplementary file 1 [file ACM2-20-15-s001.pdf]

Supplemental Table 1. Details of the setup fields, beam energy, and estimated delivery duration for the studied VMAT plans

Patient number	Field	Energy (MV)	Estimated Delivery Time (Minutes)
1	Arcs, 181º CW 179º	6	3.1
	Arcs, 179º CCW 181º	6	3.1
2	Arcs, 0º CW 179º	6	1.2
	Arcs, 179º CCW 0º	6	1.2
	Static, 345º	6	0.3
	Static, 175º	6	0.3
3	Arcs, 185º CW 179º	6	1.8
	Arcs, 179º CCW 185º	6	1.9
	Static, 0º	18	0.1
	Static, 90º	6	0.1
	Static, 175º	18	0.1
4	Arcs, 181º CW 179º	6	1.9
	Arcs, 179º CCW 181º	6	1.9
5	Arcs, 181º CW 179º	6	1.9
	Arcs, 179º CCW 181º	6	1.9
	Static, 140º	18	0.1
	Static, 180º	18	0.1
	Static, 220º	18	0.1
6	Arcs, 181º CW 179º	6	2.5
	Arcs, 179º CCW 181º	6	2.5
	Static, 90º	18	0.1
	Static, 200º	18	0.1
	Static, 345º	18	0.1
7	Arcs, 181º CW 179º	6	1.9
	Arcs, 179º CCW 181º	6	1.9
8	Arcs, 181º CW 179º	6	1.9
	Arcs, 179º CCW 181º	6	1.9
	Arcs, 335º CW 165º	6	1.0
9	Arcs, 181º CW 179º	6	1.9
	Arcs, 179º CCW 181º	6	1.9
	Arcs, 181º CW 179º	6	1.9
10	Arcs, 181º CW 179º	6	1.9
	Arcs, 179º CCW 181º	6	1.9
	Static, 0º	18	0.1

	Static, 180º	18	0.1
11	Arcs, 181º CW 179º	6	1.9
	Arcs, 179º CCW 181º	6	1.9
	Arcs, 181º CW 179º	6	1.9
12	Arcs, 181º CW 179º	6	1.9
	Arcs, 179º CCW 181º	6	1.9
13	Arcs, 181º CW 179º	6	1.9
	Arcs, 179º CCW 181º	6	1.9
14	Arcs, 181º CW 179º	6	2.5
	Static, 0º	18	0.1
	Static, 180º	18	0.1
15	Arcs, 181º CW 179º	6	2.5
	Arcs, 179º CCW 181º	6	2.5
16	Arcs, 181º CW 179º	6	3.1
	Arcs, 179º CCW 181º	6	3.1
	Static, 0º	18	0.1
	Static, 180º	18	0.2

CW: clock-wise; CCW: counter clock-wise

Supplemental Table 2. Details of the setup fields, beam energy, estimated delivery duration, and repainting number requirements, fraction number, and the use of range shifter for the studied IMPT plans

Patient number	Field (gantry, couch)	Energy range (MeV)	Estimated delivery duration (Minutes)	Maximum repainting number in one energy layer [average (min~max)]	Fraction number	Range shifter
17	150º, 180º	105.0~185.2	2.6	2 (1~6)	25	No
	150º, 0º	103.6~187.1	2.7	2 (1~6)		
18	0º, 180º	83.7~179.5	2.9	1 (1~5)	28	Yes
	180º, 180º	127.2~205.3	2.3	1 (1~3)		
19	145º, 0º	96.3~153.5	2.0	2 (1~6)	25	No
	160º, 180º	94.8~147.0	1.9	2 (1~6)		
20	180º, 270º	102.1~183.3	2.6	3 (1~6)	25	No
	150º, 0º	110.5~189.0	2.5	2 (1~6)		
21	160º, 180º	100.7 ~181.4	3.0	3 (1~14)	25	No
	155º, 0º	96.3~171.6	3.0	2 (1~10)		
22	160º, 0º	85.3 ~167.6	2.8	1 (1~7)	25	No
	160º, 180º	88.5 ~167.6	2.7	1 (1~2)		
23	150º, 0º	107.8~195.9	2.8	2 (1~5)	23	No
	180º, 270º	106.4~191.3	2.7	3 (1~5)		
24	140º, 0º	102.1~177.5	2.0	1 (1~2)	28	No
	140º, 180º	100.7~163.5	1.7	1 (1~2)		
	357º, 0º	71.3~165.6	2.5	2 (1~8)		
25	150º, 0º	94.8~195.9	3.7	6 (1~9)	25	No
	150º, 180º	96.3~198.2	3.6	6 (1~15)		
	180º, 180º	88.5~189.0	3.6	6 (1~11)		
26	165º, 180º	96.3~175.6	2.4	1 (1~5)	25	No
	155º, 0º	100.7~165.6	2.1	1 (1~2)		
27	150º, 0º	100.7~195.9	3.6	3 (1~7)	25	No
	160º, 180º	105.0~195.9	3.5	3 (1~6)		
	180º, 180º	99.2~193.6	3.6	2 (1~5)		
28	0º, 180º	82.0~175.6	2.9	3 (1~7)	23	Yes
	180º, 180º	113.2~187.1	2.5	3 (1~10)		
29	185º, 180º	91.7~177.5	3.0	1 (1~1)	28	No
	150º, 180º	94.8~195.9	3.0	1 (1~1)		

30	150º, 0º	102.1~183.3	2.6	1 (1~4)	25	No
	180º, 270º	97.8~179.5	2.7	1 (1~2)		
31	140º, 0º	83.7~179.5	3.2	1 (1~3)	25	No
	180º, 180º	76.8~173.6	3.3	1 (1~3)		
32	0º, 270º	71.3~161.5	3.7	11 (5~31)	25	No
	180º, 270º	85.3~169.6	3.5	9 (1~27)		
33	160º, 180º	85.3~175.6	2.4	1 (1~3)	25	No
	160º, 0º	78.5~171.6	2.4	1 (1~2)		
	25º, 180º	82.0~155.1	2.0	1 (1~2)		
	90º, 180º	132.1~179.5	1.2	1 (1~4)		
34	147º, 0º	105.0~189.0	2.8	5 (1~23)	25	No
	150º, 180º	113.2~187.1	2.4	5 (1~30)		
35	180º, 180º	100.7 ~198.2	2.5	1 (1~2)	25	No
	150º, 180º	114.5 ~214.0	3.1	3 (1~7)		
	152º, 0º	100.7 ~205.3	3.5	1 (1~2)		

The inhomogeneities along the proton beam path can make the OAR dose distributions particularly sensitive to uncertainties. For example, the misalignment of bony anatomy such as vertebral bodies in the proton beam path due to setup uncertainty could make the proton beams overshoot into the lungs, which are the reasons for the outliers (see Figure 2[e~f] & Supplemental Figure 2; for lung $V_{5Gy[RBE]}$, $V_{20Gy[RBE]}$ and D_{mean} in IMPT planning). On the other hand, close proximity of some organs to high dose gradient regions may make the VMAT dose distributions of these organs sensitive to setup uncertainties (see outliers in Figure 2[e]; also Supplemental Figure 3 for cord D_{max} and $D_{0.03cc}$ in VMAT planning). We also found some outliers in plan robustness evaluation may not be clinically significant; for example, the outliers in Figure 2(a) for CTV_{high} $D_{95\%}$, $D_{5\%}$-$D_{95\%}$ and D_{2cc} in VMAT were due to somewhat increased target dosing after rigid shift in S-I direction due to setup uncertainties (Supplemental Figure 1[c~e]). The protection of OARs still met the clinical requirements in the above scenario.

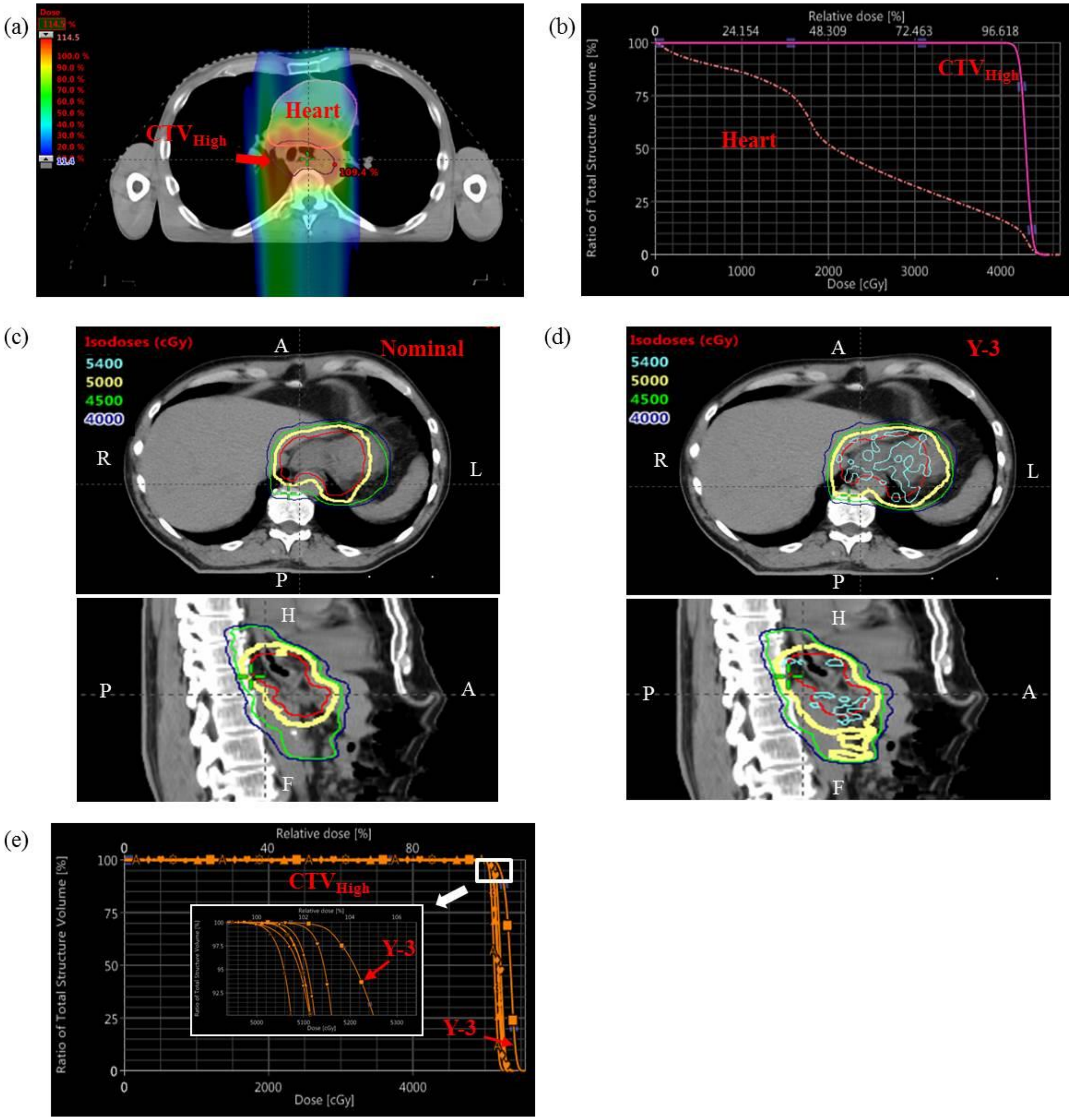

Supplemental Figure 1. Outlier analyses of DVH indices in the VMAT plan. (a) The dose distribution and spatial relation of heart and CTV_{high}. Case 1 was the outlier in heart $V_{20Gy[RBE]}$ and $V_{30Gy[RBE]}$ of IMPT plan (also see Figure 1(f)). (b) DVH curves showed the heart received high dose due to its proximity to CTV_{high}. (c) The CTV_{high}(red) dose distributions in the transversal (up) and frontal (down) views of the nominal scenario (denoted nominal). Case 2 was the outlier in CTV_{high} $D_{95\%}$ and D_{2cc} in the robustness of VMAT plan (also see Figure 2(a)). The top panel represents the axial image, versus sagittal at the bottom panel. (d) The CTV_{high} (*red*) dose distributions in the axial (up) and sagittal (down) views of the scenario after rigid shift in the superior-inferior direction with 3 mm (denoted Y-3). (e) DVH band showed Y-3 greatly increased the band width.

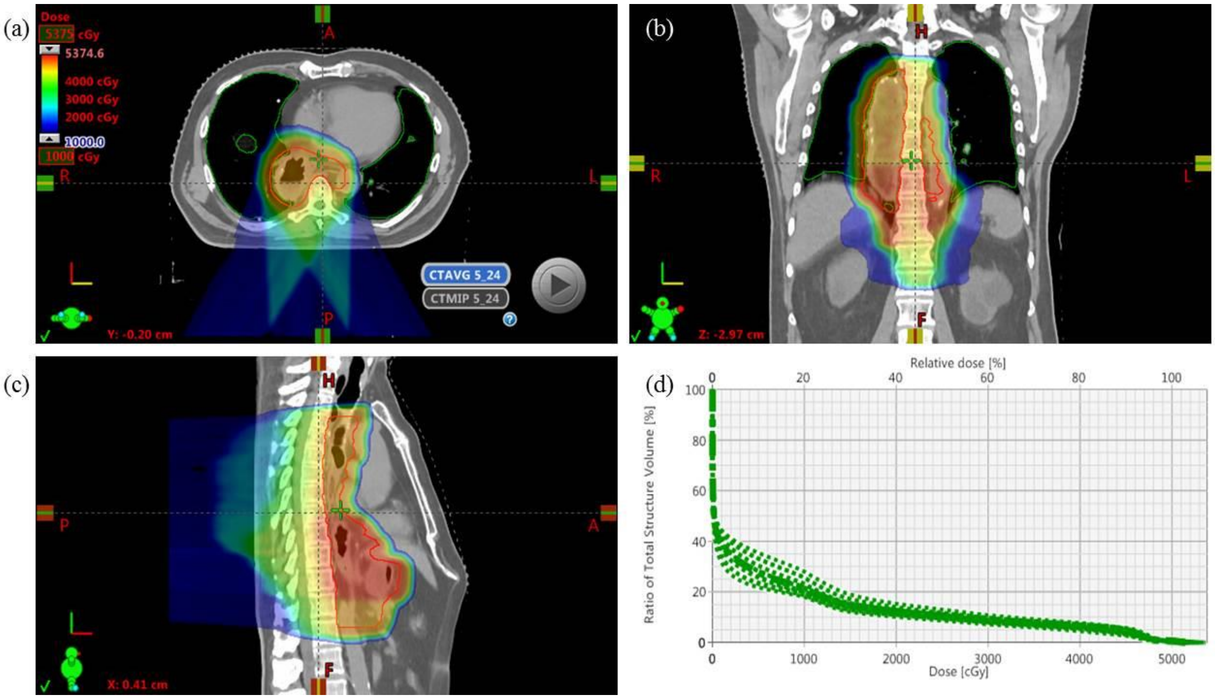

Supplemental Figure 2. Outlier analyses of lung $V_{5Gy[RBE]}$, $V_{20Gy[RBE]}$, and D_{mean} in the IMPT plans (see also Figure 2(e~f)). (a) Dose distribution is shown in the axial image in relation to the lungs (*green*) and CTV_{high} (*red*). (b) Dose distribution in the coronal images (c) Dose distribution in the sagittal images (d) DVH curves of total normal lung in 12 uncertainty scenarios. The inhomogeneities along the beam path (left posterior field) make the IMPT dose distribution of total normal lung sensitive to setup uncertainties.

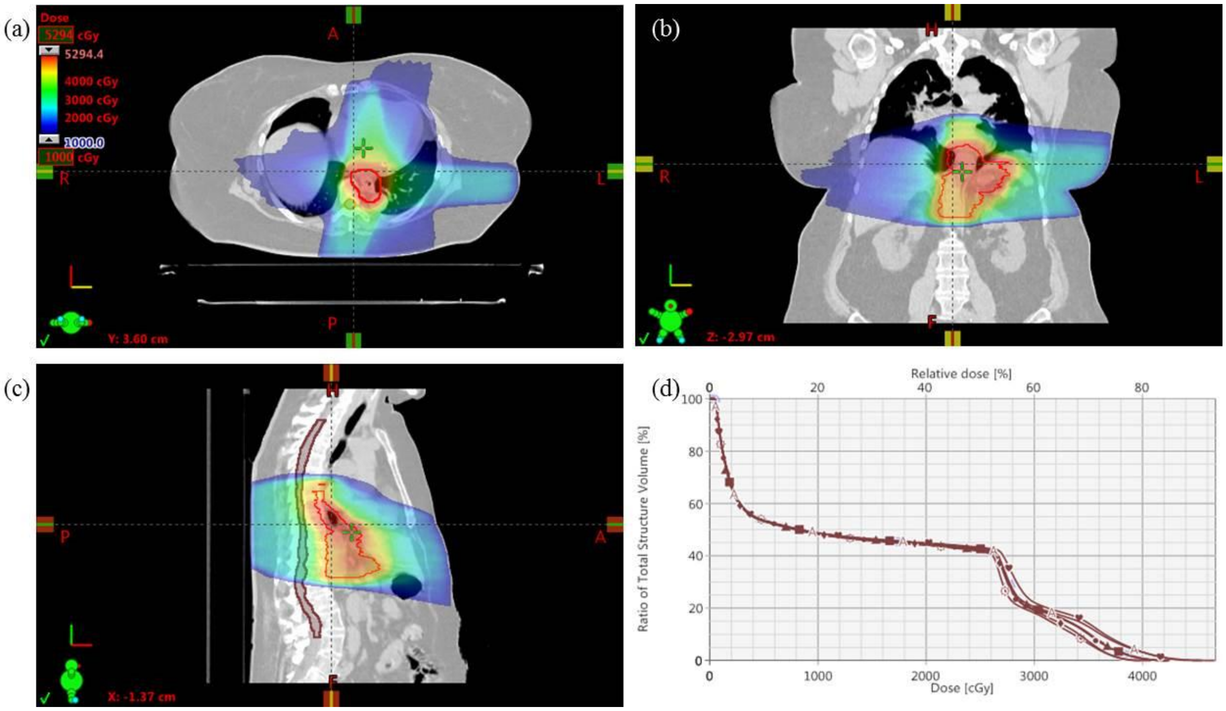

Supplemental Figure 3. Outlier analyses of cord D_{max} and $D_{0.03cc}$ in the VMAT treatment planning (see also Figure 2(e)). (a) Dose distribution in the axial images and its spatial relationship to the cord (*brown*) and CTV_{high} (*red*) are shown. (b) Dose distribution in the coronal image (c) Dose distribution in the sagittal image (d) DVH curves of the cord in six analyzed uncertainty scenarios. The cord is close to the high-dose gradient regions, which makes the cord dose distribution sensitive to setup uncertainties.
